# Case report: An index of suspicion in hyponatraemia

**DOI:** 10.11613/BM.2019.011002

**Published:** 2018-12-15

**Authors:** Marizna Barkhuizen, Mariza Hoffmann, Ekkehard WA Zöllner, Rajiv T. Erasmus, Annalise E. Zemlin

**Affiliations:** 1 Division of Chemical Pathology, National Health Laboratory Service (NHLS) and University of Stellenbosch, Tygerberg Hospital, Cape Town, South Africa; 2 Department of Paediatrics, Faculty of Medicine and Health Sciences, University of Stellenbosch, Cape Town, South Africa

**Keywords:** pseudohyponatraemia, electrolyte exclusion effect, case report, serum indices, endogenous interference

## Abstract

Serum indices can give valuable information and should be interpreted as a result. Lipaemia can influence results through different mechanisms, an important one being the electrolyte exclusion effect. A case of pseudohyponatraemia due to this is reported. A 15-year-old female with type 2 diabetes was seen for follow-up. Her biochemistry results revealed severe hyponatraemia of 118 mmol/L. Her capillary glucose concentration was 13.7 mmol/L with a corrected sodium of 122 mmol/L. A lipaemic index of 3+ (absolute value 1320) was noted, which was not flagged by the laboratory information system, as it was below the critical lipaemia limit for sodium determination. Repeated analysis of the same sample using a direct ion selective electrode method, the serum sodium concentration was 134 mmol/L (sodium corrected for glucose = 138 mmol/L). A triglyceride concentration was requested, which was severely raised (100.1 mmol/L). The electrolyte exclusion effect is an analytical phenomenon that causes falsely low electrolyte concentrations in the presence of severe lipaemia or hyperproteinaemia when using indirect analytical methods. These methods are used on many modern-day automated chemistry analysers and should be considered in a patient with asymptomatic hyponatraemia.

## Introduction

Hyponatraemia is an electrolyte abnormality that is frequently encountered in hospitalised patients (*1*). It is defined as a sodium concentration of < 135 mmol/L and is often associated with an increased morbidity and mortality (*2*). The time of onset and cause of hyponatraemia will direct management. Therefore, diagnostic algorithms provide a stepwise approach in identifying the cause of hyponatraemia (*2*). An important factor is to incorporate clinical findings and other biochemical findings when assessing a patient with hyponatraemia. The initial step is to determine whether the hyponatraemia is in fact a “true” hypotonic hyponatraemia that is in keeping with the patient’s clinical presentation.

The initial biochemistry results often do not include serum osmolality, total protein or triglyceride measurement, but assessing serum indices (haemolysis, icterus and lipaemia) could provide valuable information. *In vitro* haemolysis (evident by an increased haemolysis index) can cause a falsely low sodium concentration due to a dilutional effect from the release of intracellular content (*3*). Lipaemic samples can cause interference in a variety of analytical tests through different mechanisms. An important mechanism of interference is due to volume displacement, also known as the electrolyte exclusion effect, that affects electrolyte determination with indirect ion-selective electrode (ISE) potentiometry (*4*).

## Case Report

### Case and methods description

A 15-year-old girl attended the paediatric diabetes clinic of Tygerberg Hospital, Cape Town, South Africa for a routine check-up. On presentation she was found to have faecal loading and mild diabetic ketoacidosis (DKA). She was known to have type 2 diabetes mellitus (DM) since 2014, as well as non-alcoholic fatty liver disease, overweight and metabolic syndrome (DM, hypertension, and increased waist circumference, hypertriglyceridaemia and low high-density lipoprotein cholesterol). Management included lifestyle modification, metformin, insulin, bezafibrate and enalapril, yet her glycaemic control remained suboptimal. Poor adherence to therapy was confirmed by the patient. Right sided abdominal pain and constipation were her only presenting complaints. On physical examination she was not dehydrated or shocked. Acanthosis nigricans was noted. Lipohypertrophy, eruptive xanthomata or lipaemia retinalis were not detected. Her body mass index (BMI) was 26.0 kg/m^2^ (95^th^ percentile on UK BMI chart). Abdominal examination revealed an enlarged liver (3 cm from the costal margin in midclavicular line) and palpable faeces. The rest of the examination was unremarkable. The capillary blood glucose concentration was 22.1 mmol/L (FreeStyle Optium Neo, Abbott, Doncaster, Australia), the β-hydroxybutyrate concentration was 3.7 mmol/L (FreeStyle Optium Neo, Abbott, Doncaster, Australia). Urinalysis showed 4+ glycosuria and 4+ ketonuria (RightSign, Biotest, China). Additionally, urea, creatinine and a venous blood gas were requested. A venous blood gas could not be performed due to severe lipaemia. The blood was collected in a capillary for blood gas analysis which on visual inspection appeared clogged with severely turbid content and was therefore thought not suitable for analysis. The point of care β-hydroxybutyrate determination may be affected by triglyceride concentrations greater than 21.2 mmol/L (*5*). A mild DKA was diagnosed on the basis of hyperglycaemia > 11 mmol/L, β-hydroxybutyrate concentration > 3 mmol/L and ketonuria (*6*). The patient was treated with hourly subcutaneous insulin and recovered speedily.

Biochemical analysis was performed on a Roche Cobas 6000 analyser (Roche Diagnostics, Mannheim, Germany). Sodium concentration was determined by indirect ISE potentiometry. The triglyceride concentration was determined by an enzymatic colorimetric method. Osmometry was performed on an Advanced® Micro-Osmometer Model 3320 (Advanced instruments, Inc., Massachusetts, USA) using freeze point depression. The blood gas analyser was an ABL80 FLEX (Radiometer Medical ApS, Denmark) using a direct ISE method for electrolyte determination. The Roche Cobas 6000 analyser detects lipaemia using spectrophotometry. The lipaemia index has a semi-quantitative measuring range of 10 to 2000. The method uses diluted samples (with 0.9% sodium chloride) to measure absorbances at two different wavelengths for each analyte. The primary wavelength for lipaemia is 660 nm and secondary wavelength 700 nm (*7*).

Informed consent was obtained from the patient and her mother. The Health Research Ethics Committee of Stellenbosch University, Cape Town (South Africa) approved this case report (C18/05/011).

### Laboratory analyses

Table 1 shows the patient’s biochemistry results on the first day of admission. Her sodium concentration on admission was 116 mmol/L (sodium corrected for a glucose of 22.1 mmol/L was 123 mmol/L) with a semi-quantitative lipaemic index of 1661 (*8*). As the lipaemic index was below the instrument alert level of 2000, this result was not flagged by the laboratory information system (LIS). The treating paediatrician was contacted, because the sodium was critically low. The paediatrician was informed that pseudohyponatraemia was suspected in the light of the high lipaemic index, but the sample was insufficient for further investigations. The biochemistry tests were repeated on the same day, which again revealed a critically low sodium concentration of 118 mmol/L and semi-quantitative lipaemic index of 1320. The capillary blood glucose was 13.7 mmol/L and therefore, the corrected sodium was 122 mmol/L. After high-speed centrifugation, the sodium concentration of the infranatant was 128 mmol/L. The sodium concentration was also determined by direct ISE on a blood gas analyser, which was 134 mmol/L (sodium corrected for glucose of 13.7 mmol/L, was 138 mmol/L). The measured serum osmolality was 303 mmol/kg, with a calculated osmolarity of 269.9 mmol/L (calc osmo = 2 x Na^+^ + Urea + Glucose). This revealed an increased osmolal gap of 33.1 mmol/L (osmolal gap = measured osmolality – calculated osmolarity) (*9*). The triglyceride concentration was extremely high, with a concentration of 100.1 mmol/L after a 1:50 dilution. Acute pancreatitis was excluded as the lipase level was only mildly elevated and the patient did not have symptoms suggestive of acute pancreatitis.

**Table 1:** Patient’s biochemistry results on admission and at the follow up

**Analyte**	**Admission**	**Follow up (same day)**	**Reference interval**
Sodium, mmol/L *(indirect ISE)*	116	118	136 - 145
Sodium infranatant, mmol/L *(indirect ISE)*		128	
Sodium, mmol/L *(direct ISE)*		134	
Potassium, mmol/L	Haemolysis	Haemolysis	3.5 - 5.1
Urea, mmol/L	3.8		1.4 - 5.4
Creatinine, mmol/L	53		39 - 85
Triglycerides, mmol/L		100.1	< 1.7 (fasting)
Amylase, U/L		30	19 - 76
Lipase, U/L		35	4 - 29
**Indices**			
Haemolysis	113 (1+)	100 (1+)	
Icterus	2 (1+)	2 (1+)	
Lipaemia	1661 (4+)	1320 (3+)	
Osmolality, mmol/kg		303	275 - 295
**Point of care testing**			
Glucose, mmol/L	22.1	13.7	3.3 - 5.6
β-hydroxybutyrate, mmol/L	3.7	2.1	< 0.6
ISE - ion-selective electrode potentiometry.			

## What happened?

We describe a case of pseudohyponatraemia secondary to severe hypertriglyceridaemia in a patient with uncontrolled type 2 DM presenting with a mild DKA. The patient had severe hyponatraemia which was not solely due to hyperglycaemia causing osmotic movement of water from the intracellular space to the extracellular space (*10*). She had no signs or symptoms suggestive of severe hyponatraemia. The patient had a slight increase in measured serum osmolality, which could be due to hyperglycaemia and mild ketonaemia (*11*, *12*). Turhan *et al.* described that lipaemia did not significantly affect the measurement of serum osmolality (*13*). However, in our patient, the calculated serum osmolarity was significantly lower than the measured osmolality, which led to a high osmolal gap. This is due to calculated serum osmolarity being susceptible to lipaemia interference due to its effect on sodium determination when using indirect ISE (*13*). The analytical interference was overcome by using an alternative method (direct ISE) for sodium determination.

## Discussion

Dyslipidaemia is common in individuals with type 2 DM, with hypertriglyceridaemia being the most common lipid abnormality (*14*). Hypertriglyceridaemia is an independent risk factor for the development of cardiovascular disease, acute pancreatitis (at triglyceride concentration > 10mmol/L) and is also an important endogenous interferent in laboratory analysis (*4*, *15*-*17*).

Lipaemia is defined as the presence of high concentrations of lipoproteins in serum. A study performed in an academic centre in Iowa reported that the most common causes of lipaemia were due to type 2 DM and iatrogenic secondary to the infusion of intravenous lipid emulsions (*e.g.* total parenteral nutrition) (*18*). The time of blood sampling is also important, as sampling soon after a meal or after administration of lipid emulsions may increase the triglyceride content in serum (*19*). Lipaemia may cause interference in the analysis of many analytes, including albumin, electrolytes, lactate dehydrogenase, amylase and bilirubin (*16*). The semi-quantitative lipaemic index for interference of lipase analysis is 2000, whereas it can cause a false decrease in amylase levels above a semi-quantitative lipaemic index of 1500 and is important to consider when assessing patients with hypertriglyceridemia for possible acute pancreatitis (*20*). Therefore, a lipase would be a more suitable test, as it has less interference due to lipaemia and is also more specific for pancreatic injury (*20*–*22*).

The electrolyte exclusion effect may affect electrolyte concentrations when determined by indirect ISE or flame photometry methods. These methods involve a dilution step prior to analysis. Normal serum consists of 93% aqueous phase and 7% solids (*2*). This ratio is disturbed in the serum of patients with significant lipoprotein abnormalities, causing an increase in the solid phase fraction. A fixed volume of diluent is used during this method and leads to a higher dilution due to the change in the solid phase fraction (*2*). There are different ways to overcome this interference, the easiest of which is to repeat analysis with a direct ISE method. Electrolyte determination by this method is not affected by the electrolyte exclusion effect, because there is no dilution step prior to analysis. Bedside electrolyte analysers, including blood gas analysers use direct ISE method (*23*). However, a study by Sen *et al.* demonstrated that even direct ISE methods may be affected by lipaemia (*24*). They demonstrated a decline of 5.17% and 9.98% in the sodium concentrations determined on two different analysers using direct ISE. This study was conducted using Intralipid® and the finding should be confirmed in native hyperlipidaemic samples.

Another method to decrease lipaemia interference is through ultracentrifugation, but this equipment is not readily available in many laboratories (*25*). We attempted high-speed centrifugation of the sample and repeated analysis using indirect ISE potentiometry. The result obtained after high-speed centrifugation (128 mmol/L) was lower than the result obtained by direct ISE (134 mmol/L) and was therefore not an effective method to decrease lipaemia interference in this case. Extraction of lipoproteins through the addition of Lipoclear® (lipoprotein precipitation) is an additional method to decrease the interference due to lipaemia, but may also interfere with sodium determination (*19*, *26*). However, in our laboratory, we do not have ultracentrifugation or Lipoclear® available and if pseudohyponatraemia is suspected, we repeat the analysis using direct ISE.

Lipaemia can also cause interference with spectrophotometric methods due to the ability of lipoproteins to cause light scattering that may interfere with nephelometric or turbidimetric methods (*4*, *16*). Partitioning of analytes is another mechanism of interference leading to the movement of non-polar analytes (lipophilic analytes) into large non-polar lipoprotein particles, such as chylomicrons and very low density lipoproteins (*16*). This mechanism of interference should be considered when ultracentrifugation with removal of the lipid layer is performed, because this could lead to falsely low concentrations of analytes that are distributed in the lipid layer (*19*).

Visual inspection of samples was used for many years to detect lipaemia in serum samples (*19*). However, this method is time consuming and has wide inter-interpreter variability (*27*). With the introduction of automated pre-analytical systems, sample handling by technologists has been reduced and automated detection of serum indices became a vital part of laboratory diagnostics. The measurement of serum indices will improve reporting of results, which will have a direct effect on patient care. Management based on falsely low or elevated results due to endogenous interferences may lead to serious patient harm.

Serum indices are often included in laboratory reports at no additional cost to the patient and it is important that these indices are interpreted by the clinician. Unfortunately, many clinicians do not appreciate the importance of indices and how they affect laboratory results. Serum indices give valuable information on sample quality and may also give information about underlying conditions. In this case, the high lipaemic index alerted the clinician to after-request triglyceride concentrations. It is important to note that there is no direct correlation between the level of lipaemia and triglyceride concentration, because lipoproteins contain variable amounts of triglycerides (*19*).

As indices are determined by analysers at various wavelengths, cross-interference should also be considered when interpreting results. The haemolysis index is influenced by the degree of lipaemia due to the spectral-overlap of haemoglobin and lipaemia (*28*). The wide absorbance spectrum of lipaemia between 300 to 700 nm can contribute to the absorbance of haemoglobin, which is measured at 415 nm on the Roche Cobas 6000 (*4*, *29*). This cross-interference between serum indices can lead to inappropriate rejection of results. However, many new automated analysers are introducing correction factors to try and prevent this (*30*).

The LIS middleware rules need to be set up appropriately to flag results that may have been affected by lipaemia (and other serum indices) and cross-interference between indices. This will alert laboratory staff of the interference and allow them to implement actions to decrease the interference and to account for cross-interference before the result is transmitted to the LIS. Our patient’s sodium result was not flagged by the middleware and was transmitted to the LIS, because the current rule for lipaemia interference in sodium determination is set at a semi-quantitative index of 2000. This rule was set up according to the interference limitations specified in the package insert of the manufacturer (*30*). Roche accepts +/- 10% as the maximum allowable bias due to lipaemia for sodium determination. If we accept 134 mmol/L as the patient’s true sodium result that was determined on the blood gas analyser, this means that the acceptable lower limit due to lipaemia interference is 120.6 mmol/L (134 mmol/L - 10%). Our patient’s sodium concentration was 118 mmol/L, which is outside this lower limit at a semi-quantitative lipaemic index of 1320. Also, the difference between the true sodium value (134 mmol/L) and the maximum allowable lower bias limit (120.6 mmol/L) has significant clinical implications. This will change the severity of the patient’s hyponatraemia from mild to profound. Furthermore, this acceptance criteria for lipaemia interference is much greater than the desirable specifications for total allowable error of 0.73% according to Westgard (*31*). Therefore, the cut-off for lipaemia interference for sodium determination on the Roche Cobas analyser should be decreased from the current recommendation of 2000. The use of a +/- 10% maximum allowable bias due to lipaemia is not appropriate for sodium and the allowable bias should also take the biological variation, analytical variation and clinical implications into account. The interference thresholds set by manufacturers should be used with caution. Interference studies are often performed using Intralipid-spiked samples. The effect of Intralipid on certain analytes may differ from the effect of native lipaemia and therefore, it is recommended that lipaemic patient samples should be used for interference testing (*32*). A recent publication by von Meyer *et al.* urges manufacturers to improve the information on serum indices interference that is provided (*28*).

In conclusion, the electrolyte exclusion effect is an analytical phenomenon that causes falsely low electrolyte concentrations in the presence of severe lipaemia or hyperproteinaemia when using indirect analytical methods. This method is used on many modern-day automated chemistry analysers and should be considered in a patient with asymptomatic hyponatraemia. This case demonstrated the importance of routine reporting of serum indices and its role in patient care. Lipaemia is an important pre-analytical factor that influences the analytical phase in various ways.

## What YOU should / can do in your laboratory to prevent such errors

The thresholds for endogenous interferents determined by manufacturers should be used with caution when setting up algorithms on middleware.
Lipaemia interference and its cross interference with other indices should be identified accurately by middleware rule-based algorithms (*4*).
Appropriate analyte-specific management strategies should be in place to decrease the effect of lipaemia on results.
Measurement of sodium concentration using a direct ISE method may overcome the electrolyte exclusion effect.
Clinicians must appreciate the importance of serum indices and how they affect laboratory results.
Reflex testing of triglycerides could be considered in patients with a high lipaemic index.
Venous blood sampling should be performed after a 12 hour fasting period in non-emergency situations (*33*).
